# Test of Prosody via Syllable Emphasis (“TOPsy”): Psychometric Validation of a Brief Scalable Test of Lexical Stress Perception

**DOI:** 10.3389/fnins.2022.765945

**Published:** 2022-02-09

**Authors:** Srishti Nayak, Daniel E. Gustavson, Youjia Wang, Jennifer E. Below, Reyna L. Gordon, Cyrille L. Magne

**Affiliations:** ^1^Department of Otolaryngology – Head & Neck Surgery, Vanderbilt University Medical Center, Nashville, TN, United States; ^2^Department of Psychology, Middle Tennessee State University, Murfreesboro, TN, United States; ^3^Vanderbilt Genetics Institute, Vanderbilt University Medical Center, Nashville, TN, United States; ^4^Vanderbilt Kennedy Center, Vanderbilt University Medical Center, Nashville, TN, United States; ^5^College of Education Literacy Studies Ph.D. Program, Middle Tennessee State University, Murfreesboro, TN, United States

**Keywords:** prosody, speech, dyslexia, rhythm, phenotyping

## Abstract

Prosody perception is fundamental to spoken language communication as it supports comprehension, pragmatics, morphosyntactic parsing of speech streams, and phonological awareness. A particular aspect of prosody: perceptual sensitivity to speech rhythm patterns in words (i.e., lexical stress sensitivity), is also a robust predictor of reading skills, though it has received much less attention than phonological awareness in the literature. Given the importance of prosody and reading in educational outcomes, reliable and valid tools are needed to conduct large-scale health and genetic investigations of individual differences in prosody, as groundwork for investigating the biological underpinnings of the relationship between prosody and reading. Motivated by this need, we present the Test of Prosody via Syllable Emphasis (“TOPsy”) and highlight its merits as a phenotyping tool to measure lexical stress sensitivity in as little as 10 min, in scalable internet-based cohorts. In this 28-item speech rhythm perception test [modeled after the stress identification test from Wade-Woolley (2016)], participants listen to multi-syllabic spoken words and are asked to identify lexical stress patterns. Psychometric analyses in a large internet-based sample shows excellent reliability, and predictive validity for self-reported difficulties with speech-language, reading, and musical beat synchronization. Further, items loaded onto two distinct factors corresponding to initially stressed vs. non-initially stressed words. These results are consistent with previous reports that speech rhythm perception abilities correlate with musical rhythm sensitivity and speech-language/reading skills, and are implicated in reading disorders (e.g., dyslexia). We conclude that TOPsy can serve as a useful tool for studying prosodic perception at large scales in a variety of different settings, and importantly can act as a validated brief phenotype for future investigations of the genetic architecture of prosodic perception, and its relationship to educational outcomes.

## Introduction

Prosody, the pattern of stress and intonation in speech, is one of the most overlooked features of language, despite its many functions in human communication. During discourse, listeners need to extract relevant information from continuous speech streams, interpreting each word, phrase, and sentence that is spoken, and integrating each utterance into the broader context of the conversation. Listeners can benefit from prosody at various levels of this complex process. For example, prosodic cues such as patterns of stressed and unstressed syllables facilitate the listener’s ability to identify boundaries between words (e.g., Mattys and Samuel, 1997) and bolster speech segmentation during infants’ language development (Jusczyk, 1999). At the sentence level, syllabic stress patterns across words form a rhythmic structure that facilitates the resolution of lexically ambiguous syllable sequences (Dilley and McAuley, 2008). Prosody also helps listeners parse the syntactic cues of sentences (Pynte and Prieur, 1996), understand the speaker’s emotions (Besson et al., 2002), and distinguish between questions and statements (Wales and Taylor, 1987). In discourse situations, speakers often use prosodic cues (e.g., pitch accent) to attract listeners’ attention toward relevant information (e.g., Cutler et al., 1997).

Converging evidence also suggests a link between reading acquisition and sensitivity to the lexical stress cues provided by the pattern of stress and unstressed syllables (Holliman, 2016). Moreover, individual differences studies on typically developing young readers show that children’s performance on a variety of novel lexical stress perception tasks accounts for unique variance in reading ability, even after controlling for phonological awareness (Holliman et al., 2008, 2017; Clin et al., 2009) which is itself a long-established predictor of successful early reading development (Adams, 1990). There is recent evidence that prosodic sensitivity becomes an even stronger predictor of word reading than phonological awareness as children grow older, especially when tested with multisyllabic words (Enderby et al., 2021). Prosodic sensitivity may be particularly relevant for mastering reading of multisyllabic words (e.g., 3 or more syllables) because lexical stress patterns in these words is less predictable. Further, as multisyllabic words become more frequent in reading materials for older readers (i.e., in later elementary grades) the frequency of multi/poly-morphemic words also increases (e.g., *cultivation*) compared to mono-morphemic words (e.g., *grow*), making phonemic decoding strategies harder to rely upon during reading. Similarly, studies in a sample of adults with a wide range of reading ability found that lexical stress task performance continues to predict individual differences in multisyllabic word reading (Chan and Wade-Woolley, 2018) as well as readers’ ability to correctly apply punctuation to written language (Heggie and Wade-Woolley, 2018), which facilitates sentence construction, reading comprehension, and reading fluency (Schwanenflugel et al., 2004).

Despite its importance for a range of language functions, the biological mechanisms of prosody remain largely unexplored. Large-scale etiologic studies, such as genome-wide association studies (GWAS), may open promising doors to better understanding the biological basis of prosody and its potential biological overlap with reading skills. Crucially, GWAS of complex (non-Mendelian) traits such as speech and reading would require very large sample sizes in order to reproducibly capture genetic associations of interest (Deriziotis and Fisher, 2017). Recent advances in the genetic architecture of dyslexia in a sample of over one million participants (Doust et al., 2021, *MedRXiv*) are promising in their utilization of genotyped cohorts in conjunction with questionnaire data and the ability to demonstrate shared genetic bases between reading disorder and other health traits. The goal of extending these lines of research to other speech, language, and reading traits highlights the need for coordinated efforts toward collecting and meta-analyzing large-scale data in cohorts that have been able to link language-related traits to genotypes. [e.g., GenLang consortium (genlang.org): Eising et al., 2021, *BioRXiv*, Lancaster et al., *in prep.*; International Stuttering Project (theinternationalstutteringproject.com): Polikowsky et al., 2021; Shaw et al., 2021].

The genomic basis of prosody necessitates the development and validation of scalable, internet-based tools that are well-suited for larger scale population health studies, in part by maximizing automation and minimizing burdens on administration time and effort. Such “brief” phenotypes of other cognitive-related traits (e.g., psychiatric conditions, non-verbal cognition, or health history) can be deployed at large scales when they characterize or measure traits as precisely as possible using brief methods (e.g., short questionnaires, existing medical chart notes, scales, or observational assessments) and are already widely represented in biobanks. They have been utilized extensively by recent GWAS studies to map phenotype-genotype relationships (*see* Watanabe et al., 2019). Yet in contrast to the brief self-reports utilized for phenotyping many health traits in clinical settings (e.g., depressive symptoms), or self-reports of history of speech/language/reading problems, a linguistic trait such as prosodic perception needs to be tested more objectively, disentangled from participants’ meta-awareness about these abilities. This is in line with calls for brief, reliable, and scalable tools to enable large-scale GWAS of complex cognitive traits (Coleman, 2021).

Ideally, approaches for measuring speech-language traits such as prosody perception would take the form of validated, reliable, short, and easy-to-administer online tasks. Further, for easier scalability and reduced administration times, such measures will ideally be automatically scored and not rely on manual scoring by a trained clinician or experimenter. Accordingly, we present here a novel internet-based measure of prosodic sensitivity to lexical stress patterns that is also relevant for future investigations of its genetic architecture and relationship with reading skill. The current study aimed to develop a measure of lexical stress perception for internet-based use at scale (e.g., for genetics investigations), modeled after an existing lexical stress task with high reliability. The stress identification subtest of a prosodic awareness task (Wade-Woolley et al., 2012; Chan and Wade-Woolley, 2018; Heggie and Wade-Woolley, 2018) was selected as the model, due to its relationship with reading skill, and its good internal consistency in adults (Cronbach’s α = 0.83: Heggie and Wade-Woolley, 2018; Cronbach’s α = 0.81: Chan and Wade-Woolley, 2018). In the stress identification task, participants were asked to listen to pre-recorded multi-syllabic words of varying lengths (two to five syllables: Heggie and Wade-Woolley, 2018; four or five syllables in Chan and Wade-Woolley, 2018) and asked to identify the syllable that was the main beat, emphasis, or stress for each word (e.g., *KNOWledge*) or the stress position (e.g., “the first syllable”). Responses were made orally to the task administrator.

The current work builds on prior success with measuring lexical stress, to improve the quality of speech rhythm (prosody) perception phenotypes for large-scale behavioral and genetic studies. While reliable phenotyping is currently available in biobanks and electronic health records for many health, cognitive, and psychiatric traits, speech-language and reading traits are generally underrepresented in these large-scale databases and are often superficially phenotyped (Raghavan et al., 2018). Efforts are therefore underway to mine available speech-language phenotypes in existing electronic health records using sophisticated computational methods such as automated pipelines (e.g., for identifying cases with Developmental Language Disorder: Walters et al., 2020) and machine learning (e.g., for imputing stuttering cases: Pruett et al., 2021). Both approaches aim to decrease manual effort (e.g., clinical chart review) toward achieving the scales required for genetic investigations. Motivated by similar goals, we present a new tool for measuring lexical stress at scale.

A useful and valid measure of speech rhythm perception should be expected to have good predictive validity for difficulties with speech-language and reading skills based on the literature. Reading-related impairments in phonological processing are a key diagnostic criterion for dyslexia (Morris et al., 1998), and these phonological deficits are present at both the segmental (phonemic) and suprasegmental (i.e., prosodic) levels, as demonstrated by the poorer sensitivity to prosodic cues and non-verbal auditory rhythm cues in individuals with dyslexia (Goswami et al., 2010; Leong et al., 2011). Therefore, we hypothesized that lexical stress test performance would be related to a history of dyslexia and developmental speech-language disorders or difficulties.

At the point of intersection between prosody, musical rhythm, and speech-language-reading impairments, we also expect lexical stress test performance to be related to musical rhythm ability. Recent work shows that rhythm deficits are commonly found in children with speech, language, and reading disorders (Ladányi et al., 2020), and that musical rhythm and speech rhythm processing are supported by shared neural mechanisms such as synchronization of neural oscillations to external stimuli, and sensorimotor coupling (Fiveash et al., 2021). Similarly, individual differences in speech rhythm perception are associated with musical rhythm perception abilities (Hausen et al., 2013; Morrill et al., 2015; Magne et al., 2016), pointing to domain-general sensitivity to rhythm extending to both musicality and speech (Fiveash et al., 2021).

Here, we report on the design, development, and validation of our **T**est **o**f **P**rosody via **SY**llable emphasis (TOPsy). The test takes 10 min to complete; is automatically scored; and can be administered over the internet or in lab settings. TOPsy is different from the stress identification task it was modeled after (Wade-Woolley et al., 2012; Chan and Wade-Woolley, 2018; Heggie and Wade-Woolley, 2018) in two important ways: (1) counterbalancing for syllabic stress position, since initially stressed items are much more frequent in English and therefore may elicit higher performance on a lexical stress perception task: (2) counterbalancing for lexical item length (# of syllables), as more complex lexical items may capture lexical stress perception differently. To examine the potential effects of lexical stress position and lexical length on lexical stress perception, we report on the test items’ factor structure and reliability metrics. As an additional proof-of-concept, we examine TOPsy’s predictive validity for difficulties with speech-language, reading, and musical rhythm, which are all known to be associated with prosodic perception (Goswami et al., 2010; Magne et al., 2016; Fiveash et al., 2021). Specifically, we conduct logistic regressions to examine whether TOPsy performance predicts significantly higher odds of presence of speech-language therapy in childhood, presence of a Dyslexia diagnosis, or self-reported inability to clap to a musical beat (a previously validated self-report measure: Niarchou et al., 2021, *BioRXiv*), as proxies of speech-language, reading, and musical rhythm difficulties, respectively.

## Materials and Methods

### Participants

Data from N = 2508 adults ages 18-88 were used in the present study from a larger ongoing internet-based study of the biological basis of rhythm, known as the Vanderbilt Online Musicality Study. Participants were recruited into the larger study between December 2019 and October 2020 from five sources: (i) ResearchMatch.org, (ii) newsletters and research mailing lists at Vanderbilt University Medical Center, (iii) Reddit.com (i.e., Ask Me Anything events on the subdomains *reddit.com/r/AskScience* and *reddit.com/r/Science*), (iv) Facebook advertising, and (v) social media sharing (i.e., participants were given shareable materials upon completion of the task and encouraged to share the study on social media or by other means). Most participants were from the United States (e.g., Facebook and ResearchMatch advertisements targeted United States-based individuals only), but participants outside the United States were also welcomed to complete the survey, for example if they found the post through Reddit.com or through social media. The landing page of the survey specified that English-speaking adults were invited to participate in the study. Further, the consent form stated that individuals should self-identify as an “adult, fluent English speaker without hearing loss” to participate.

Ethical approval was obtained from the Vanderbilt University Institutional Review Board, and participants gave written informed consent prior to participation. Study data were managed and stored using Research Electronic Data CAPture (REDCap) tools (Harris et al., 2009). Of the total N = 3,258 individuals who enrolled and initiated participation in the study, data from N = 2,508 individuals were included in the present analyses after filtering out participants with unusable or incomplete data, and excluding participants who did not pass the attention checks within the speech prosody perception test (see *Procedures* for details). An additional participant was excluded as an invalid case due to scoring zero on all tasks of interest, significantly below chance performance.

### Measures

#### Test of Prosody via Syllable Emphasis

TOPsy is a novel test of speech rhythm perception, modeled after the Stress Identification task (Wade-Woolley et al., 2012; Chan and Wade-Woolley, 2018; Heggie and Wade-Woolley, 2018). In each of the 35 trials in TOPsy, participants are presented with recordings of a spoken word along with a visual aid showing the syllabic segmentation of the word (e.g., VI | TA | MIN). Participants were asked to identify the syllable that holds the main stress or emphasis in the word, selecting from options consisting of all available syllables (e.g., for “vitamin” participants selected from the options “vi,” “ta,” and “min”, by clicking the corresponding button). Note that while all syllables were presented as possible response options, the last syllable of any given word never carried the main stress, by design. Each word presented is stressed on either the first (e.g., VI-ta-min), the second (e.g., Tech-NO-logy), the third (e.g., en-ter-TAIN-ment), or the fourth (e.g., com-mu-ni-CA-tion) syllable. Across the 35 items of TOPsy, stimuli were experimentally manipulated both in terms of which syllable was stressed (either the first, second, third, or fourth syllable) and how many total syllables the word contained (3, 4, or 5 syllables).

Embedded within the stimulus presentation block was an additional “attention check” item. In the attention check item, participants heard an auditory message such as “for this question, select the third option”; and were excluded from analyses if they responded incorrectly.

The sequence of presentation of the items was determined in advance to ensure a balanced mix of number of syllables and syllable stress positions within lexical items throughout the task (e.g., no two consecutive stimuli had both the same word length and same lexical stress position). This pre-determined item presentation sequence was the same for all participants, to avoid varying item-position effects across participants, which could influence the psychometric validity of the test.

Participants saw a message throughout the test reminding them to respond based on how the speaker in the recording was speaking the words, and not how they themselves would speak it. The speaker, who is a Speech-Language Pathologist, was instructed to use lexical stress patterns representative of standard American English, and the lexical stress patterns were confirmed through acoustic analysis of the speech stimuli (see Supplementary Materials for acoustic analysis details). Prior to the stimulus presentation block, participants were asked to complete a short headphone test (detailed in *Procedures* below).

##### Item Development

Across items, TOPsy controls for word frequency, and aims for a balanced representation of the number of syllables and syllable stress position of lexical items. Words presented in TOPsy were selected from the English Lexicon Project database (Balota et al., 2007^1^). All words were classified as nouns and were between three to five syllables in length. Further, words were selected to have a minimum frequency in English. Frequencies in Balota et al. (2007) were based on the Hyperspace Analog to Language (HAL) corpus (Lund and Burgess, 1996), consisting of approximately 131 million words gathered in 1995. TOPsy words all had a Log HAL frequency of ≥ 5, indicating log transformed frequency norms based on the HAL corpus (see Supplementary Materials for details of item characteristics).

##### Speech Stimuli

Speech stimuli were recorded by an adult female native English speaker using an AKG cardioid condenser microphone (AKG P220) placed 1.5 – 2 ft from the speaker. The AKG microphone rested on a shockmount during recording, to inhibit any noise from vibrations. Speech stimuli were recorded in a quiet environment, inside a sound isolation booth (Whisper Room, Inc.). Each word was recorded 3 times and the clearest recording was later selected for development of TOPsy. Recordings were filtered for any background environmental noise using Audacity. All recordings were edited such that the sound clip was the length of the word being spoken without blank space on either side. To confirm that syllabic stress assignments were indeed consistent with acoustic features of stressed patterns (i.e., stress identification scored as “correct” answers were objectively correct), we conducted phonetic analyses of each item. These analyses confirmed that vowels of all stressed syllables were longer and louder than vowels of unstressed syllables, regardless of syllabic stress position within words (see Supplementary Materials for details and results of phonetic analyses).

#### Syllable Counting Test

A syllable counting test was included to account for variability in TOPsy scores due to phonological processing skills such as syllabification, by including syllable counting performance as a relevant covariate in all analyses. During the test, participants listened to auditory recordings of single multi-syllabic words (3, 4, or 5 syllables long, similar to TOPsy items), but with no visual cues or scaffolding. Similar to TOPsy items, participants were asked to respond by clicking on a button corresponding to options “3”, “4”, or “5”, to indicate the number of syllables in the word.”

#### Questionnaires of Demographic and Speech/Language/Rhythm History

Participants reported their age, race, ethnicity, and sex at birth. They also reported their highest level of education with a 6-item multiple choice question ranging from “Less than high school education” to “Doctorate or equivalent degree.” Participants answered three additional questions about their reading and speech-language history, with three response options (Yes, No, I don’t know): “Have you ever been diagnosed with dyslexia?,” “Did you get speech-language therapy as a child?,” and “Are you a native English speaker?”.

Participants were asked about their musical beat synchronization ability through a single question: “can you clap in time with a musical beat?” with three response options (Yes, No, I’m not sure). Although this is a single-item measure of musical rhythm ability, self-reports to this question have been shown to map on well to behavioral measures of musical rhythm ability such as musical beat synchronization and beat-based rhythm discrimination, including discriminating both simple and complex (syncopated) rhythms (Niarchou et al., 2021, *BioRXiv*). Further, Niarchou and colleagues have successfully used this approach to phenotyping musical beat synchronization in the first GWAS of musical rhythm. Beat synchronization is a key musical rhythm skill that recruits sensory (i.e., auditory), motor, and executive function systems (*see* Repp and Su, 2013, *for review*). Beat synchronization skills have been previously found to correlate with perceiving, responding to, and processing speech rhythms (Lidji et al., 2011; Woodruff Carr et al., 2014; Lagrois et al., 2019). A valid test of speech rhythm should therefore show a relationship with beat synchronization.

### Procedures

#### Internet-Based Data Collection

Following the landing page describing the details of the study, participants provided informed consent before proceeding to the study tasks. The consent form and all online questionnaires and tasks were implemented in REDCap, a secure web platform for building and managing research databases and surveys.

All participants were instructed to use headphones for sound stimuli at the start of the experiment. A 4-trial headphone test (Woods et al., 2017) was used to ensure audio fidelity, in which participants heard a series of 3 tones and indicated which tone was played the softest. During the calibration test, stimuli could be replayed by participants to make sure the sound on their computer or phone was calibrated well, but during the task this feature was disabled so that participants could not play the sound file in each trial of TOPsy or the Syllabification task more than once. While the headphone test is described in the manuscript to give a complete view of the study procedures, no one was excluded based on their headphone test results.

The Syllable Counting test and TOPSy were then administered as described above (sections “Test of Prosody via Syllable Emphasis” – “Syllable Counting Test”). Participants then completed the self-report questionnaire (section “Questionnaires of demographic and related speech/language/rhythm history”). All participants were then directed to a feedback page where they could report problems and give feedback to the study investigators. Finally, participants were provided with feedback on their performance for TOPsy. Participation in the larger study also involved three components not in the scope of the current paper: an additional brief questionnaire (prior to the headphone test), another auditory task, and optional offline DNA collection via mail-in saliva sampling.

#### Data Quality Checks and Cleaning Process

Each of the primary sections of the online study contained an attention-check item designed to check that participants were paying attention to task instructions (e.g., “For this item, please choose “Disagree”). If a participant did not accurately complete the attention check item within TOPsy, they were filtered out of the analyses reported here. Near the end of the study, participants were also asked to give a yes-no rating to the quality of their data (i.e., “Do you believe that your questionnaire responses are accurate and that your data is usable?”). Participants who responded “No” to this question were excluded from all analyses. Participant data was also excluded from analyses if they reported experiencing technical difficulties while completing the study. For example, those who reported a loss of audio output during at least two trials had their data from the affected task excluded from analyses. Furthermore, completed consent forms were manually reviewed for repeated instances of name-age combinations, to identify participants who completed the study more than once. In these cases, data was only included for a participant’s first valid completion of the study.

### Analyses

First, descriptive statistics for demographic and performance variables were analyzed. Exploratory factor analysis (EFA) was then conducted to understand the underlying factor structure that best explains variance in TOPsy performance. Three model fit indices were used to evaluate overall model fit: root mean square error of approximation (RMSEA; Steiger, 1990), Tucker–Lewis index (TLI; Tucker and Lewis, 1973), and Bayesian information criterion (BIC; Schwarz, 1978). RMSEA values between 0.05 and 0.08 denote acceptable fit while values lower than 0.05 express good fit (Hu and Bentler, 1998). TLI values between 0.90 and 0.95 are considered good fit while values greater than 0.95 are considered excellent (Hu and Bentler, 1998). No cut-off has been proposed for BIC but models with lower BIC values are generally preferred. As the chi-square goodness-of-fit test is not recommended when the sample size is very large (Bentler and Bonett, 1980), chi-square values are provided for information only.

Based on EFA results and other considerations such as phonetic analyses of stimuli, some items were deleted, and remaining analyses were informed by follow-up EFA results on the final TOPsy items retained. Analyses on the final TOPsy items included computing reliability metrics, and predictive validity of TOPsy with respect to speech-language and reading difficulties, and musical rhythm skills. Analyses were conducted using *R 4.0.2* (R Core Team, 2021) and *JASP* (JASP Team, 2020).

## Results

### Descriptive Statistics

**FIGURE 1:**
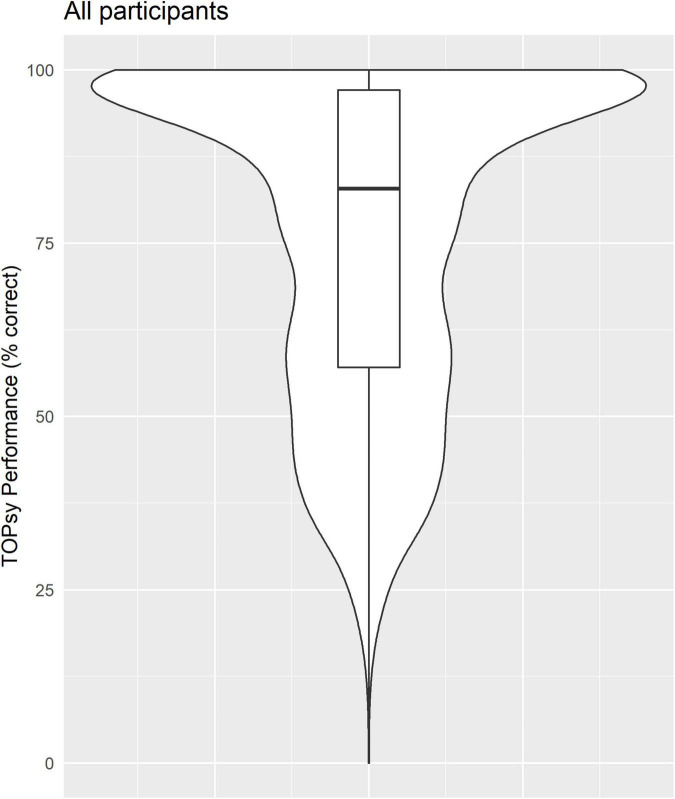
Variability in speech rhythm perception in the sample, as measured by TOPsy scores, is illustrated as percentage correct on the 35-item test. Scores ranged from 11.4% to 100%, with a median score of 82.9%. Box plot indicates interquartile ranges around the median.

Table 1 shows descriptive statistics and distributions for all demographic, self-report, and TOPsy performance variables. In our sample, ∼20% responded correctly to all 35 TOPsy items. Figure 1 illustrates the distribution of TOPsy performance in the sample.

**TABLE 1:** Descriptive statistics.

	Skew	Kurtosis	Mean	SD	Median	Min	Max
Age (yrs)			42.79	15.98	39	18	88
TOPsy (%)	−0.65	−0.86	76.26	22.97	82.9	11.4	100
Syllable counting (%)	−2.73	9.28	93.85	12.04	100	10	100

					**Yes**	**No**	**I don’t know**

Native speaker?					2730	133	3
Dyslexia?					60	2422	26
Speech language Therapy?					313	2167	28
Can clap with a musical Beat?					2333	53	122

					**Male**	**Female**	**Undisclosed**

Sex					683	1813	12

		** < High School**	**High school**	**Some College**	**Bachelors**	**Master’s**	**Doctorate**

Educational attainment		5	105	491	874	740	293

*N = 2508.*

### Factor Structure Underlying Test Items

EFA was conducted using principal axis factoring extraction to explore the factor structure of the 35 items. As we did not expect the sub-dimensions of the test to be fully independent from each other, we used an oblique Promax rotation to optimize the factor solution. The results of both Kaiser-Meyer-Olkin test of sampling adequacy (KMO = 0.972) and Bartlett’s test of sphericity [*χ^2^*(595) = 27416.01, *p* < 0.001] suggested the data were suitable for factor analysis. The scree plot, parallel analysis, and Kaiser’s criterion (eigenvalues > 1.0) suggested that a two-factor model was optimal (Supplementary Figure 1). In addition, all model fit indices for the two-factor solution showed acceptable-to-good fit values (Supplementary Table 3). By contrast, the one-factor solution was not acceptable in terms of TLI (0.86). The two factors explained 32.6% of the variance in total TOPsy scores. A close inspection of the item factor loadings indicated that all initially stressed items (N = 12) showed the strongest loadings on factor 1 while most of the non-initially stressed items (20 out of 23) had strongest loadings on factor 2, regardless of specific syllable stress position (see Supplementary Table 4 for the factor loadings of each item). However, 6 items had low factor loadings (all < 0.38), and one non-initially stressed item (“repository”) had a factor loading > 0.4 on factor 1. These 7 items were thus removed and a second EFA was conducted [KMO = 0.965; *χ^2^*(378) = 21296.29, *p* < 0.001] on the remaining 28 items with the same extraction and rotation methods as before.

Results of the second EFA also suggested an optimal 2-factor solution (Supplementary Figure 2), explaining 33.8% of the variance, and satisfactory goodness of fit indices (Supplementary Table 5). All initially stressed items (N = 12) showed strongest factor loadings on factor 1 while all non-initially stressed items (N = 16) showed strongest loadings on factor 2. All items presented factor loading > 0.4 on only one of the two factors and cross-loading ≤ 0.2 (Supplementary Table 6 for the factor loadings of the final set of items). In the rest of the manuscript, we refer to the construct underlying factors 1 and 2 as *head-stress sensitivity* and *tail-stress sensitivity*, respectively.

Note that based on goodness of fit indices, a one-factor solution performs acceptably, with a two-factor solution being ideal. However, since the two factors presented a significant moderate-to-strong correlation (*r* = 0.76, *p* < 0.01), the use of TOPsy total scores by researchers interested in an overall measure of lexical stress sensitivity would also be acceptable. Therefore, further reliability and validity analyses were conducted using the set of 28 items selected for TOPsy, as well as separately in *head-stressed* and *tail-stressed* factors.

### Reliability

Internal consistency between the 28 TOPsy items was analyzed using the *Psych* package for *R* (Revelle, 2020). The overall measure showed high reliability (Cronbach’s α = 0.92) with inter-item correlations within the ideal range (average *r* = 0.29; see Supplementary Materials for strength of correlations between each item and every other item). Internal consistency is thought to be ideal when average *r* is between 0.20 and 0.40 (Piedmont, 2014), indicating that items consistently measure the same construct, but also elicit sufficient unique variance in performance and are distinct from each other. Further, the median inter-item correlation (*r* = 0.28) was similar to the average *r*, reinforcing the overall homogeneity of the scale. Item-whole correlations (i.e., correlations between scores on each item and total scores, corrected for scale reliability and item overlap) ranged from *r* = 0.41 to *r* = 0.65. Further, item-whole correlations comparing each item with test scores if this item were *dropped*, indicated a similar range from *r* = 0.40 to *r* = 0.62).

The two subscales that are supported by the factor structure also showed high reliability (head-stress sensitivity α = 0.88; tail-stress sensitivity α = 0.87) Additional reliability metrics for the *head-stressed* factor showed the following results: average *r* = 0.37; median *r* = 0.37; item-whole correlations ranging from *r* = 49 to *r* = 67, and from *r* = 0.46 to *r* = 0.62 when each item was dropped. Similarly, for the *tail-stressed* items: average *r* = 0.30; median *r* = 0.30; item-whole correlations ranging from *r* = 0.46 to *r* = 0.65, and from *r* = 0.44 to *r* = 0.60 when each item was dropped.

### Predictive Validity

#### Predictive Validity for History of Language and Reading-Related Difficulties

Next, logistic regressions were conducted to examine predictive validity of TOPsy scores for language, reading-related, and musical rhythm skills, both in the overall set of items, and separately in *head-stressed* and *tail-stressed* items. In our sample, N = 60 reported a dyslexia diagnosis, N = 313 reported having received speech-language therapy as a child, and N = 53 reported not being able to clap in time with a beat. There was minimal overlap between those who reported dyslexia and speech-language therapy (N = 16).

**FIGURE 2:**
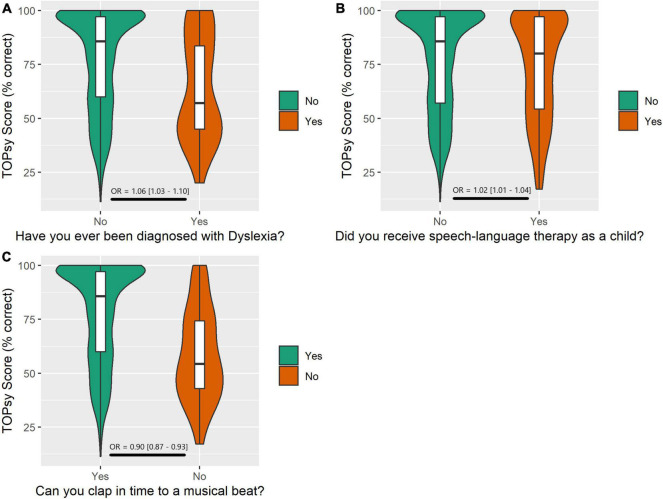
Comparative distributions of lexical stress perception scores on the 28-item TOPsy are illustrated. The right side of plots **(A–C)** illustrate distributions in those who reported a dyslexia diagnosis (i.e., reading difficulties), receiving speech-language therapy in childhood (i.e., speech-language difficulties), or not being able to clap in time with a beat (i.e., musical rhythm difficulties) respectively. The left side of each plot illustrates distributions in those who reported no difficulties on self-report questions.

**FIGURE 3:**
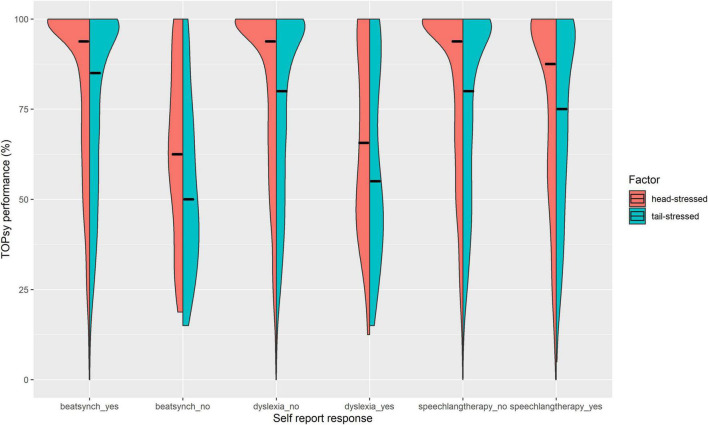
Comparative distributions of lexical stress perception scores on initially stressed items (“head-stressed”; *n* = 16 items) and non-initially stressed items (“tail-stressed”; *n* = 12 items), by responses to self-report questions. Median TOPsy scores (%) are marked by horizontal black lines. Within each construct, logistic regressions showed that lexical stress perception significantly predicted higher odds of reporting difficulties with musical rhythm, reading, and speech-language skills. As illustrated, TOPsy performance distributions were noticeably different between yes and no responses, with less of a ceiling effect, and lower medians in individuals reporting difficulty synchronizing with a beat; a dyslexia diagnosis; and a history of speech-language therapy. Lower TOPsy performance most strongly predicts odds of difficulty with beat synchronization (musical rhythm). Differences in median scores and distributions between *head-stressed* vs. *tail-stressed* items are relatively stable across all self-report responses and constructs.

Logistic regression analyses showed that lower TOPsy scores were significantly correlated with increased odds of dyslexia [Figure 2; OR = 1.06 [CI: 1.03 – 1.10], *p* = 0.00021, McFadden’s pseudo *R*^2^ (Adj.) = 0.034], holding age, educational attainment, sex, native speaker status, and syllabification skills constant. On average, a 1-point decrease in TOPsy score was associated with a 6% increase in odds of reporting dyslexia. Further, regression analyses showed that lower scores on *head-stressed* and *tailed-stressed* items separately correlated with increased odds of dyslexia (Figure 3), accounting for the same covariates [*head-stressed*: OR = 1.11 [CI = 1.05 – 1.18], *p* = 0.00059, McFadden’s pseudo *R*^2^ (Adj.) = 0.029; *tail-stressed*: OR = 1.09 [CI = 1.04 – 1.15], *p* = 0.00063, McFadden’s pseudo *R*^2^ (Adj.) = 0.030]. The above analyses were conducted on the subset of participants that responded either “Yes” or “No” to the dyslexia and native speaker questions, and who chose to report sex (N = 2467).

Similarly, a second regression model with the same covariates showed that lower TOPsy scores were significantly correlated with increased odds of having received speech-language therapy as a child [Figure 2; OR = 1.02 [CI: 1.01 – 1.04], *p* = 0.0018, McFadden’s pseudo *R*^2^ (Adj.) = 0.022]. On average, a 1-point decrease in TOPsy scores was associated with a 2% increase in odds of reporting speech-language therapy. Further, lower scores on head-stressed and tailed-stressed items also separately, and significantly, predicted increased odds of speech-language therapy [Figure 3; *head stressed:* OR = 1.04 [CI = 1.02 – 1.08], *p* = 0.0018, McFadden’s pseudo *R*^2^ (Adj.) = 0.022; *tail-stressed*: OR = 1.04 [CI = 1.01 – 1.06], *p* = 0.0066, McFadden’s pseudo *R*^2^ (Adj.) = 0.022]. The above analyses were conducted on the subset of participants that responded either “Yes” or “No” to the speech-language therapy and native speaker questions, and who chose to report sex (N = 2464).

#### Predictive Validity for Musical Rhythm Skills

Logistic regression analyses showed that higher TOPsy scores were significantly correlated with decreased odds of reporting an inability to synchronize with a musical beat [Figure 2; OR = 0.90 [CI: 0.87 – 0.93], *p* < 0.0001, McFadden’s pseudo-*R*^2^ (Adj.) = 0.085]. On average, a 1-point increase in TOPsy scores was associated with a 10% decrease in the odds of responding “No” (vs “Yes”) to the beat synchronization question. Further, lower scores on head-stressed and tailed-stressed items also separately predicted increased odds of beat synchronization abilities (Figure 3), accounting for the same covariates (*head-stressed*: OR = 0.85 [CI = 0.80 – 0.91], *p* < 0.0001, McFadden’s pseudo *R*^2^ (Adj.) = 0.059; *tail-stressed*: OR = 0.85 [CI = 0.80 – 0.90]), *p* < 0.0001, McFadden’s pseudo *R*^2^ (Adj.) = 0.085). The above analyses were conducted on the subset of participants that responded either “Yes” or “No” to the beat synchronization and native speaker questions, and who chose to report sex (N = 2372).

## Discussion

This study reports results from a novel test of speech rhythm sensitivity, the Test of Prosody via Syllable Emphasis (TOPsy), administered in a large internet-based cohort. Specifically, we reported details of test design and development, analysis of the underlying factor structure, and an improved shortened version of the test. We also reported reliability metrics and predictive validity for musicality and communication traits known to be related to impairments in speech rhythm, specifically difficulties with musical rhythm (beat synchronization), and history of reading disorder and speech-language therapy. We further showed that the ability to detect syllable stress in lexical items that are initially and non-initially stressed are not equal. Further, task performance loaded best onto a two-factor solution consisting of a “head-stress sensitivity” factor (initially stressed items) and “tail-stressed sensitivity” factor (non-initially stressed items, regardless of specific stress position). Further reliability analyses confirmed high internal consistency within the sets of specific test items that loaded onto the head-stress and tail-stress factors.

The dissociation between head-stress sensitivity and tail-stress sensitivity in our data is particularly interesting given that initially stressed and non-initially stressed words have different frequencies of occurrence and developmental trajectories in English. Carlson et al. (1985) reported that less than 20% of words were weak-initial in a corpus of the 10,000 most common word forms in English. Likewise, Cutler and Carter (1987) analyzed a corpus of 190,000 English words and found that about 90% of polysyllabic content words started with a stressed syllable. This probabilistic difference between weak-initial and strong-initial words in English has been proposed to bootstrap language acquisition early on (Cutler and Norris, 1988). In line with this hypothesis, Jusczyk (1999) reported that 7.5-months-old infants showed a sensitivity to strong-initial words but not weak-initial words while 10.5-month-olds were sensitivity to both. Thus, infants appear to develop sensitivity to the most common stress pattern (i.e., strong initial syllable) first. Further, children at age 7 are still thought to be acquiring adult-like production of weak-strong lexical stress pattern, whereas children as young as 3 years could already produce lexical items with strong-weak patterns marked by adult-like duration and intensity (Ballard et al., 2012). To aid future use of TOPsy in younger populations, we have provided typical age of acquisition for each test item.

Reliability analyses results showed excellent internal consistency (Cronbach’s α ranging from 0.87 to 0.92 depending on items included), in line with the original Stress Identification Task that TOPsy was modeled after, which reported high Cronbach’s α (0.81 to 0.83) in previous studies (Chan and Wade-Woolley, 2018; Heggie and Wade-Woolley, 2018). Additional reliability metrics such as mean and median inter-item correlations were found to be within the ideal range, confirming that TOPsy items measured the speech rhythm sensitivity construct consistently homogenously, while also remaining distinct (i.e., eliciting some unique variance in performance).

As hypothesized, predictive validity analyses in the final 28-item version of TOPsy showed that lower speech rhythm perception scores predicted increased risk of history of developmental speech-language disorders, and inability to synchronize with a musical beat. The associations between TOPsy scores and speech-language and musical rhythm abilities are consistent with the broader literature on relationships between linguistic and musical rhythm perception and processing (Gordon et al., 2011; Hausen et al., 2013; Magne et al., 2016; Fotidzis et al., 2018); and with reports of relative rhythm impairments in a host of developmental speech-language disorders (Ladányi et al., 2020).

Further, TOPsy scores predicted risk for dyslexia (i.e., reading related difficulties), consistent with previous findings that prosodic sensitivity – particularly speech rhythm sensitivity – explains variability in reading and literacy skills in children (Whalley and Hansen, 2006; Wood, 2006; Holliman et al., 2008, 2012) and adults (Kitzen, 2001). These results are further consistent with a growing body of research showing that children and adults with dyslexia perform less accurately than typical peers on speech rhythm perception tasks, suggesting that underdeveloped speech rhythm sensitivity may be a prevalent feature of dyslexia (Goswami, 2011; Leong et al., 2011; Jiménez-Fernández et al., 2015). Understanding individual differences in prosodic sensitivity and its biological associations with reading can inform future efforts to understand mechanisms of reading-related disorders, as well as clinical and educational interventions to promote skilled reading, in line with calls for improving reading interventions and instruction strategies (Sawyer, 2010; Nippold, 2015). Importantly, here we find converging results with previous literature using a brief phenotyping approach to measure speech rhythm perception, with test administration and automatic scoring time kept under 10 min.

Two additional advantages of TOPsy are: (1) It minimizes the confounding role of executive function deficits (e.g., difficulties with working memory, flexible attentional shifting, or inhibitory skills) which are often comorbid with reading and language disorders. For example, the task does not require holding and operating on stimuli in working memory (e.g., comparing two stimuli), rather requires a direct and immediate response to speech stimuli one at a time. Similarly, there are no rule changes throughout the task, and therefore minimal demands on flexible switching or updating skills; and the task is not timed or speeded, minimizing inhibitory control or motor control demands. Overall, these features make the test more accessible to individuals with speech-language or reading disorders, who may have comorbid executive function difficulties. (2) It captures perceptual sensitivity to lexical stress specifically, in contrast to global prosody measures which may conflate sensitivity to intonation, affect, focus, or pragmatic style.

The current study has a few limitations and raises questions for future research. *First*, tests of construct validity (e.g., by comparing performance on TOPsy with existing measures of prosody perception in the same sample) were not utilized in the current work. This was largely due to a lack of standardized or validated tests for measuring lexical stress (or prosody more generally) in large internet-based cohorts, and limited psychometric data available for existing tests, making it difficult to test construct validity in a meaningful way. Future work should thus test construct validity of TOPsy, in lab-based studies, in relation to other lexical stress tests.

Relatedly, while TOPsy shows promising predictive validity for self-reported speech/language, reading, and musical beat synchronization difficulties, logistic regressions found relatively weak associations between TOPsy performance and these self-reported measures. Future studies should include behavioral measures or standardized assessments of reading-related, speech-language, and musical rhythm abilities to robustly evaluate TOPsy’s predictive validity for these constructs. Planned future directions include (a) evaluating predictive validity for silent reading performance, via follow-up efforts in the large internet-based cohort described in this work; (b) evaluating predictive validity for standardized assessments of reading-related skills in an independent lab-based sample; (c) incorporating lab-based or scalable internet-based behavioral measures of musical rhythm perception. We further encourage the research community to assess TOPsy’s predictive validity for psychological constructs known to be associated with speech rhythm perception (or prosody more broadly), in large-scale and/or collaborative studies.

*Second*, the current study does not report test-retest reliability due to testing being limited to one time-point, but longitudinal study designs will allow us to expand psychometric analyses of TOPsy in the future. *Third*, given that a substantial proportion of our sample performed at ceiling, future work should further explore item-level metrics such as item discrimination and item-difficulty to gain insights on which items are the most informative to capture wide variability in speech rhythm perception abilities. While the current study focused on an intentionally broad range of individuals, future studies should aim to validate TOPsy within clinical speech-language populations (e.g., individuals with Developmental Language Disorder, stuttering, dyslexia, or aphasia) whose speech-language phenotypes have been extensively characterized, in order to establish the test’s utility and relationship to specific impairments of speech, language, and reading in these populations. Additionally, since only about 5% of our sample comprised non-native speakers of English, future investigations validating TOPsy for non-native speakers, or English language learners, would also expand the potential for future investigations of speech rhythm perception.

Future work should also consider effects of dialectical variations on speech rhythm perception, something that TOPsy design attempts to mitigate with two strategies for directing participants’ attention to the rhythm of the word they heard in each trial: (a) reminding participants to respond based on the stress pattern in the audio recordings, not based on their own assigned stress pattern; and (b) with an “attention check” where the accurate response to an item presented approximately mid-way, can only be arrived at by listening to the recording (i.e., to prevent individuals from responding only based on the written item). However, it remains possible that for some subset of individuals, the lexical stress for a few recorded items were inconsistent with lexical stress patterns in their dialect. This potential incongruence could in theory affect variance on individual items by placing an additional burden on executive control (having to inhibit internal representations of lexical stress) or working memory (having to remember a less familiar lexical stress pattern). Relationships between prosody and dialect variation can be understood through broader sociolinguistics questions (Holliday, 2021). Similarly, a limitation is that speaker variability in the stimuli could in theory affect lexical stress perception in this test. Therefore, future iterations of this line of research (including additional validation studies of TOPsy) could include stimuli pronounced by several speakers, and several tokens of the same word, in order to test for generalization of speech rhythm perception.

Based on the promising results reported herein, we argue that wide adoption of TOPsy will be useful as a reliable and valid speech rhythm or prosody phenotype. The test may be particularly relevant to researchers interested in understanding individual differences or perceptual systems related to speech-language, reading, or musical rhythm abilities (and related disorders), as it would allow pragmatic inclusion of speech rhythm perception in large-scale epidemiological cohorts. Individual differences studies across a broad range of abilities, as compared to investigations focused on clinical samples, can provide more precision in both characterizing specific speech-language and reading-related impairments, as well as in designing intervention efforts (National Academies of Science Engineering and Medicine, 2016). Importantly, TOPsy can be adopted as a reliable and valid measure in large-scale efforts to understanding the biological bases of prosodic perception, such as its heritability (e.g., using twin and family based methods), and its genetic architecture (e.g., using GWAS methods).

In line with calls to conduct large-scale health investigations of speech and language traits (Raghavan et al., 2018), TOPsy can enable collaborative interdisciplinary research on the biology and health relevance of individual differences in lexical stress perception, a key aspect of prosodic skills. Among other opportunities, it opens doors for researchers in many fields (e.g., epidemiology, computational genetics, sociolinguistics, education, social work, global health) to incorporate the study of prosody into important ongoing investigations of a wide range of speech and language traits with implications for human development outcomes.

## Data Availability Statement

The raw data supporting the conclusions of this article will be made available by the authors, without undue reservation.

## Ethics Statement

The studies involving human participants were reviewed and approved by Vanderbilt University Institutional Review Board. The participants provided their written informed consent to participate in this study.

## Author Contributions

CM, RG, YW, and DG designed the study materials. SN, YW, and DG collected the data. SN and YW prepared the data for analysis. SN, DG, and CM analyzed the data and wrote and revised the manuscript. JB and RG consulted on study design, data collection, analyses, and the manuscript. All authors contributed to the article and approved the submitted version.

## Author Disclaimer

The content is solely the responsibility of the authors and does not necessarily represent the official views of the funders.

## Conflict of Interest

The authors declare that the research was conducted in the absence of any commercial or financial relationships that could be construed as a potential conflict of interest.

## Publisher’s Note

All claims expressed in this article are solely those of the authors and do not necessarily represent those of their affiliated organizations, or those of the publisher, the editors and the reviewers. Any product that may be evaluated in this article, or claim that may be made by its manufacturer, is not guaranteed or endorsed by the publisher.

## Footnotes

1
https://elexicon.wustl.edu
