# Supplementary material for: Test of Prosody via Syllable Emphasis (“TOPsy”): Psychometric Validation of a Brief Scalable Test of Lexical Stress Perception
Source: Front Neurosci. 2022 Feb 9;16:765945. doi: 10.3389/fnins.2022.765945 (PMC8864136; doi:10.3389/fnins.2022.765945)
Supplement: Supplementary file 1 [file Data_Sheet_1.docx]

Supplementary Material

# Item Characteristics

Supplementary Table S.1 shows syllable lengths, syllable emphasis positions, and log frequencies for each test item. Age of acquisition (AoA) for each item is also reported, from the English Lexicon Project database. AoA is provided to inform future developmental applications of TOPsy but was not a factor in item selection.

**Supplementary Table S.1**

| *TOPsy Item Characteristics* | | | | |
| --- | --- | --- | --- | --- |
| **Word** | **NSyll** | **Stress Pos** | **Log Freq HAL** | **AoA** |
| library | 3 | 1 | 11.63 | 4.95 |
| passenger | 3 | 1 | 8.35 | 6.65 |
| vitamin | 3 | 1 | 8.24 | 5.42 |
| continent | 3 | 1 | 8.21 | 8.35 |
| discussion | 3 | 2 | 11.48 | 7.40 |
| example | 3 | 2 | 11.84 | 7.39 |
| horizon | 3 | 2 | 8.40 | 8.30 |
| inventor | 3 | 2 | 8.22 | 8.00 |
| literature | 4 | 1 | 9.93 | 9.90 |
| laboratory | 4 | 1 | 9.72 | 8.72 |
| secretary | 4 | 1 | 9.61 | 7.75 |
| television | 4 | 1 | 10.02 | 4.11 |
| agriculture | 4 | 1 | 8.61 | 10.32 |
| category | 4 | 1 | 9.74 | 8.15 |
| relationship | 4 | 2 | 10.42 | 9.11 |
| technology | 4 | 2 | 11.62 | 9.06 |
| development | 4 | 2 | 11.78 | 8.63 |
| ability | 4 | 2 | 10.95 | 8.84 |
| environment | 4 | 2 | 11.17 | 9.85 |
| motivation | 4 | 3 | 8.52 | 10.60 |
| registration | 4 | 3 | 10.10 | 10.62 |
| education | 4 | 3 | 10.98 | 6.70 |
| entertainment | 4 | 3 | 9.72 | 7.39 |
| veterinary | 5 | 1 | 7.58 | 7.84 |
| federalism | 5 | 1 | 5.71 | u/a |
| refrigerator | 5 | 2 | 7.52 | 4.11 |
| repository | 5 | 2 | 8.19 | 14.83 |
| vocabulary | 5 | 2 | 7.94 | 6.74 |
| accelerator | 5 | 2 | 8.55 | 10.27 |
| manufacturer | 5 | 3 | 9.56 | 10.79 |
| university | 5 | 3 | 12.38 | 10.72 |
| flexibility | 5 | 3 | 8.78 | 9.65 |
| organization | 5 | 4 | 10.76 | 9.16 |
| civilization | 5 | 4 | 9.07 | 10.00 |
| communication | 5 | 4 | 10.70 | 8.47 |
| *Note*. NSyll = number of syllables; Stress Pos = position of stressed syllable; Log Freq HAL = log transformed lexical frequencies for each test item, drawn from the *Hyperspace Analog to Language* corpus of over 200 million words (Lund & Burgess, 1996). Age of acquisition (AoA) for each item is also reported, from the English Lexicon Project database (Balota et al., 2007). | | | | |

# Acoustic Analysis of TOPsy Items

Relative to unstressed syllables, stressed syllables usually have a combination of longer duration, greater intensity, and higher pitch, with duration potentially being the most consistent cue (Okobi, 2006). To confirm that lexical stress assignments were indeed consistent with acoustic features of stressed patterns (i.e., stress identification scored as “correct” answers were objectively correct), we conducted phonetic analyses of each item. To automatically align the audio recordings with their corresponding orthographic, the Montreal Forced Aligner (McAuliffe et al., 2017) was used in combination with a pronunciation dictionary and acoustic models pre-trained on the English corpus LibriSpeech (Panayotov et al., 2015). The resulting phone-level segmentation was visually checked for accuracy of alignment in Praat (Boersma & Weenink, 2001) and manually corrected if necessary. Acoustic measurements of the vowels in each word included duration (ms), measured from onset to offset of the vowel, peak intensity (dB), and peak f0 (Hz).

These acoustic measures were also used to calculate normalized pairwise variability index (nPVI: Grabe and Low, 2008) values for duration, peak intensity, or peak f0 for each word. The nPVI provides a useful measure to compare stress contrastivity between words that include a strong-weak vs. weak-strong pattern. It is calculated using the formula nPVI = 100 x {(v1-v2)/[(v1+v2)/2]}, where v1 and v2 are measures of duration, peak intensity, or peak f0 of vowels within a target pair of adjacent syllables. Therefore, a positive nPVI value indicates first-syllable stress, while a negative nPVI value indicates second-syllable stress. To determine whether production of stress contrasts could be affected by the number of syllables and/or stress position, analyses focused on comparing pairs of adjacent stressed and unstressed syllables. For initially stressed words, vowels in the first and second syllables were used, while for non-initially stressed words (i.e., stress located on either the second, third or fourth syllable), vowel measurements were extracted from the stressed syllables and the preceding unstressed syllable.

These analyses confirmed that vowels of all stressed syllables were longer and had greater intensity than vowels of unstressed syllables, regardless of syllabic stress position within words. In line with previous studies on isolated word production (Arciuli & Slowiaczek, 2007), pitch appears to be a less reliable stress cue (*see* Table S.2).

## Supplementary Table S.2

| *Acoustic Cues of the Target Vowels* | | | | | | | | | | |
| --- | --- | --- | --- | --- | --- | --- | --- | --- | --- | --- |
|  | Stress location | Duration | | | Peak Intensity | | | Peak f0 | | |
|  |  | S1 | S2 | nPVI | S1 | S2 | nPVI | S1 | S2 | nPVI |
| 3-syllable words | 1 | 122 | 78 | 44.0 | 62.0 | 59.6 | 3.9 | 234.9 | 195.3 | 18.4 |
|  | 2 | 54 | 124 | -78.7 | 59.2 | 60.9 | -2.8 | 212.6 | 210.4 | 1.0 |
|  |  |  |  |  |  |  |  |  |  |  |
| 4-syllable words | 1 | 115 | 67 | 52.7 | 64.0 | 59.9 | 6.6 | 230.6 | 198.0 | 15.2 |
|  | 2 | 77 | 124 | -46.8 | 59.2 | 63.0 | -6.2 | 205.3 | 205.5 | -0.1 |
|  | 3 | 53 | 116 | -74.6 | 62.1 | 62.8 | -1.1 | 209.6 | 213.3 | -1.7 |
|  |  |  |  |  |  |  |  |  |  |  |
| 5-syllable words | 1 | 90 | 46 | 64.7 | 63.6 | 58.3 | 8.7 | 211.4 | 196.9 | 7.1 |
|  | 2 | 65 | 98 | -40.5 | 58.2 | 63.0 | -7.9 | 202.0 | 221.5 | -9.2 |
|  | 3 | 80 | 93 | -15.0 | 60.2 | 64.9 | -7.5 | 206.7 | 208.2 | -0.7 |
|  | 4 | 60 | 119 | -65.9 | 56.6 | 62.3 | -9.6 | 199.0 | 212.2 | -6.4 |
| *Note.* nPVI = normalized pairwise variability index (Grabe & Low, 2008) | | | | | | | | | | |

# Exploratory Factor Analyses (EFA)

The first EFA was conducted on all 35 items initially included in the TOPsy. Figure S.1, Table S.3 and Table S.4 present the Scree plot, goodness-of-fit indices, and factor loadings, respectively. The second EFA was conducted based on a revised set of 28 items. Items were removed prior to the second EFA if they loaded relatively weakly onto either factor (< |0.4|). One additional item, “repository” was further removed to maintain the test’s consistent pattern of non-initially stressed items (i.e., *tail-stressed*) loading best onto Factor 1 and initially stressed items (i.e., *head-stressed*) loading best onto Factor 2. Scree plot, goodness-of-fit indices and factor loadings are presented in Figure S.1, Table S.5, and Table S.6, respectively.

**Supplementary Figure S.1**

| *Scree Plot Obtained from the Exploratory Factor Analysis of the 35 TOPsy Items* |
| --- |
| 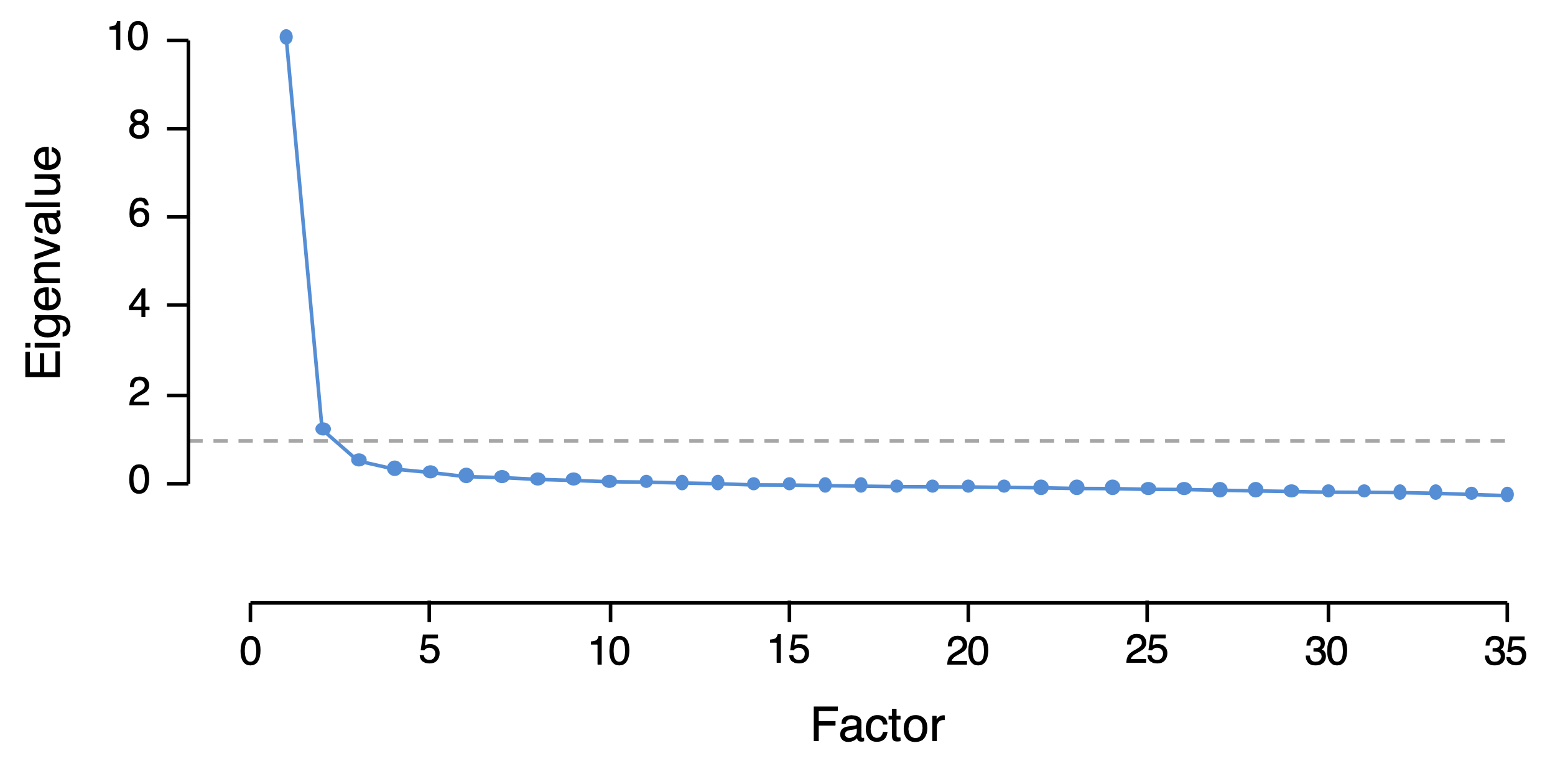 |
| *Note.* Scree plot showing eigenvalues for the 35 factors. The dashed gray line indicates the Kaiser's criterion of eigenvalues > 1.0. |

**Supplementary Table S.3**

| *Goodness of Fit Indices for the Exploratory Factor Analysis of the 35 TOPsy Items* | | | | | | | | |
| --- | --- | --- | --- | --- | --- | --- | --- | --- |
| Model | Chi-squared | df | | RMSEA | RMSEA 90% confidence | | TLI | BIC |
| 1 factor | 4129.57*** | 560 | | 0.051 | 0.049 - 0.052 | | 0.86 | -254.35 |
| 2 factors | 2141.51*** | 526 | | 0.035 | 0.033 - 0.037 | | 0.93 | -1976.25 |
| *Note.* *** p < 0.001. | | |  | | |  | | |

**Supplementary Table S.4**

| *Factor Loadings from the Exploratory Factor Analysis of the 35 TOPsy Items* | | |
| --- | --- | --- |
| Item | Factor 1 | Factor 2 |
| Category | **0.75** | -0.17 |
| Continent | **0.64** | -0.14 |
| Television | **0.70** | -0.13 |
| Veterinary | **0.64** | -0.04 |
| Secretary | **0.66** | -0.02 |
| Library | **0.57** | 0.02 |
| Vitamin | **0.50** | 0.04 |
| Agriculture | **0.63** | 0.03 |
| Laboratory | **0.57** | 0.05 |
| Federalism | **0.61** | 0.09 |
| Passenger | **0.42** | 0.11 |
| Literature | **0.58** | 0.12 |
| Discussion | 0.13 | **0.40** |
| Ability | 0.08 | **0.41** |
| Inventor | 0.08 | **0.42** |
| Environment | 0.16 | **0.44** |
| Example | 0.17 | **0.44** |
| Registration | 0.01 | **0.47** |
| Entertainment | 0.04 | **0.50** |
| Technology | 0.19 | **0.51** |
| Development | 0.18 | **0.52** |
| Civilization | -0.11 | **0.55** |
| Flexibility | 0.05 | **0.56** |
| University | -0.13 | **0.57** |
| Communication | 0.01 | **0.60** |
| Education | -0.03 | **0.60** |
| Organization | -0.10 | **0.65** |
| Motivation | -0.17 | **0.72** |
| Repository | **0.43** | 0.08 |
| Refrigerator | 0.37 | 0.15 |
| Relationship | 0.34 | 0.30 |
| Vocabulary | 0.33 | 0.24 |
| Accelerator | 0.32 | 0.34 |
| Horizon | 0.18 | 0.38 |
| Manufacturer | 0.10 | 0.37 |
| *Note.* Bold indicates factor loadings equal or greater than the absolute value of 0.4 | | |

**Supplementary Figure S.2**

| *Scree Plot Obtained from the Exploratory Factor Analysis of the 28 Retained TOPsy Items* |
| --- |
| 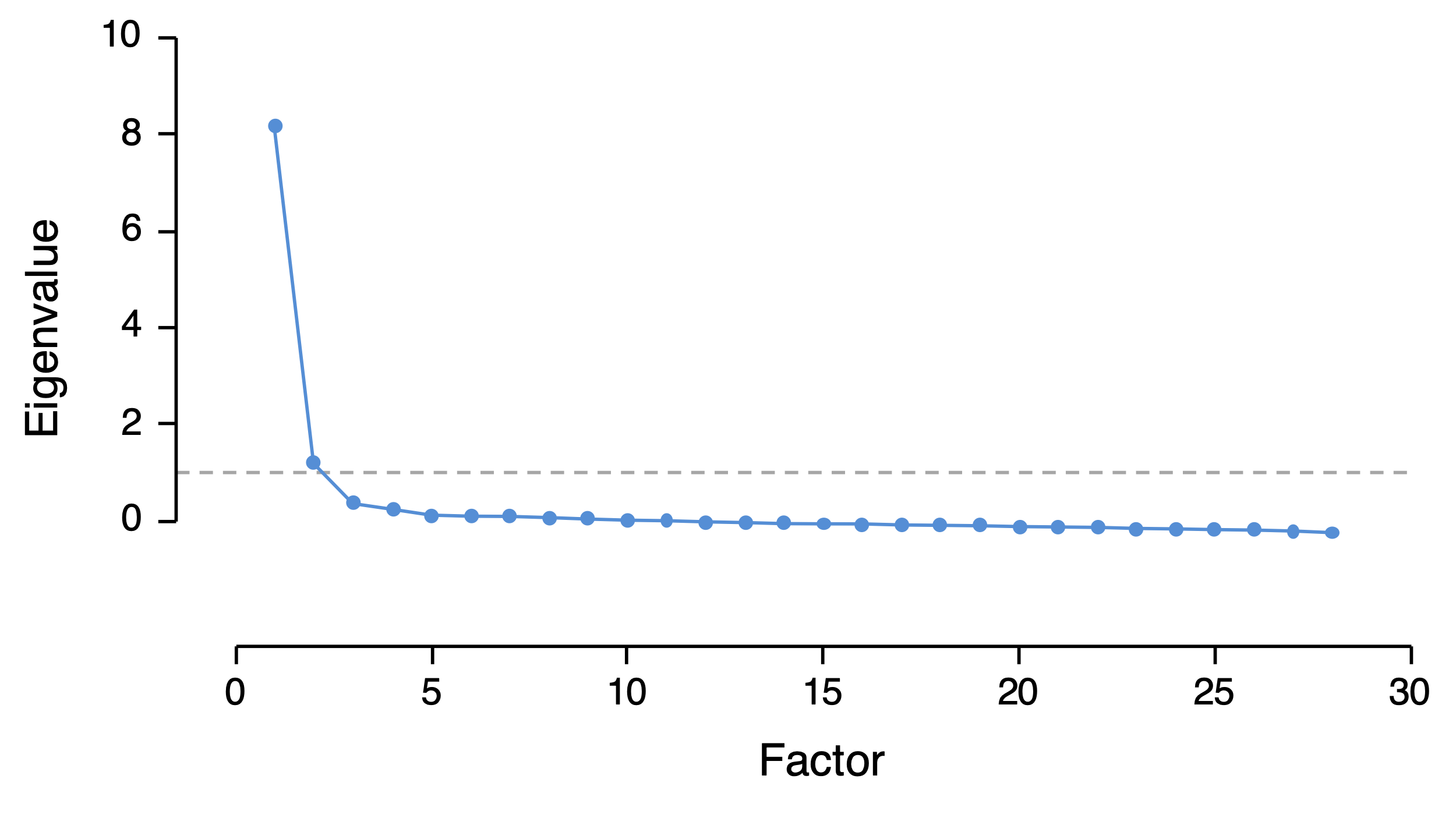 |
| *Note.* Scree plot showing eigenvalues for the 28 factors. The dashed gray line indicates the Kaiser's criterion of eigenvalues > 1.0. |

**Supplementary Table S.5**

| *Goodness of Fit Indices for the Exploratory Factor Analysis of the 28 Retained TOPsy Items* | | | | | | | | |
| --- | --- | --- | --- | --- | --- | --- | --- | --- |
| Model | Chi-squared | df | | RMSEA | RMSEA 90% confidence | | TLI | BIC |
| 1 factor | 3172.87*** | 350 | | 0.057 | 0.055 - 0.059 | | 0.85 | 432.92 |
| 2 factors | 1247.88*** | 323 | | 0.034 | 0.032 - 0.036 | | 0.95 | -1280.7 |
| *Note.* *** p < 0.001. | | |  | | |  | | |

**Supplementary Table S.6**

| *Factor Loadings from the Exploratory Factor Analysis of the 28 Retained TOPsy Items* | | | | | |
| --- | --- | --- | --- | --- | --- |
| Item | |  | Factor 1 | | Factor 2 |
| Category | |  | **0.72** | | -0.13 |
| Continent | |  | **0.62** | | -0.10 |
| Television | |  | **0.68** | | -0.10 |
| Veterinary | |  | **0.62** | | -0.01 |
| Secretary | |  | **0.65** | | 0.01 |
| Library | |  | **0.57** | | 0.04 |
| Vitamin | |  | **0.51** | | 0.05 |
| Agriculture | |  | **0.63** | | 0.05 |
| Laboratory | |  | **0.57** | | 0.07 |
| Federalism | |  | **0.59** | | 0.12 |
| Passenger | |  | **0.40** | | 0.12 |
| Literature | |  | **0.57** | | 0.14 |
| Discussion | |  | 0.13 | | **0.40** |
| Ability | |  | 0.06 | | **0.41** |
| Inventor | |  | 0.07 | | **0.41** |
| Environment | |  | 0.16 | | **0.44** |
| Example | |  | 0.18 | | **0.44** |
| Registration | |  | 0.03 | | **0.45** |
| Entertainment | |  | 0.05 | | **0.49** |
| Technology | |  | 0.20 | | **0.50** |
| Development | |  | 0.17 | | **0.52** |
| Civilization | |  | -0.08 | | **0.54** |
| Flexibility | |  | 0.06 | | **0.55** |
| University | |  | -0.12 | | **0.56** |
| Communication | |  | 0.03 | | **0.59** |
| Education | |  | -0.01 | | **0.59** |
| Organization | |  | -0.07 | | **0.65** |
| Motivation | |  | -0.14 | | **0.71** |
| Repository |  | | | Removed | |
| Refrigerator |  | | | Removed | |
| Relationship |  | | | Removed | |
| Vocabulary |  | | | Removed | |
| Accelerator |  | | | Removed | |
| Horizon |  | | | Removed | |
| Manufacturer |  | | | Removed | |
| *Note.* Bold indicates factor loadings equal or greater than the absolute value of 0.4 | | | | | |

# Inter-Item Correlations

Average inter-item correlations, i.e., the degree to which each item correlated with all other items, was found to be *r* = 0.29. Further, average inter-item correlations of head-stressed items (the first factor identified through EFA) were *r =* 0.37, and tail-stressed items (the second factor identified through EFA) were *r =* 0.30. Figure S.2 illustrates relative strengths of correlations between each item and all other items included in the 28-item TOPsy.

**Supplementary Figure S.3**

*Inter-Item Correlations in TOPsy Performance*


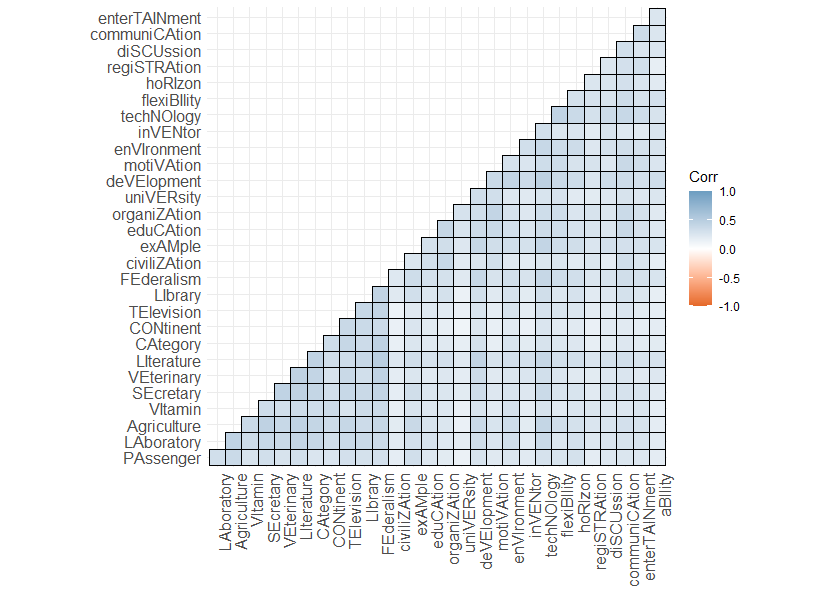


*Note.* Inter-Item Correlations between each of 28 TOPsy items and all other items. Capitalized syllables indicate lexical stress positions in recorded auditory stimuli. Here, head-stressed lexical items (i.e., initially stressed) are represented adjacent to each other, as are tail-stressed items (i.e., non-initially stressed).

References

Arciuli, J., & Slowiaczek, L. M. (2007). The where and when of linguistic word-level prosody. *Neuropsychologia*, *45*(11), 2638-2642.

Balota, D. A., Yap, M. J., Cortese, M. J., Hutchison, K. A., Kessler, B., Loftis, B., Neely, J. H., Nelson, D. L., Simpson, G. B., & Treiman, R. (2007). The english lexicon project. *Behavior Research Methods*, *39*(3), 445–459. <https://doi.org/10.3758/BF03193014>

Boersma, Paul & Weenink, David (2021). Praat: doing phonetics by computer. Version 6.1.52. http://www.praat.org/

Grabe, E. & Low, E. (2008). Durational variability in speech and the Rhythm Class Hypothesis. In C. Gussenhoven & N. Warner (Ed.), *Laboratory Phonology 7*(pp. 515-546). Berlin, New York: De Gruyter Mouton. <https://doi.org/10.1515/9783110197105.2.515>

Lund, K., & Burgess, C. (1996). Producing high-dimensional semantic spaces from lexical co-occurrence. *Behavior Research Methods, Instruments, and Computers*, *28*(2), 203–208. https://doi.org/10.3758/BF03204766

McAuliffe, M., Socolof, M., Mihuc, S., Wagner, M., & Sonderegger, M. (2017, August). Montreal Forced Aligner: Trainable Text-Speech Alignment Using Kaldi. In *Interspeech* (Vol. 2017, pp. 498-502).

Okobi, A. O. (2006). Acoustic correlates of word stress in American English (*Doctoral dissertation, Massachusetts Institute of Technology*).

Panayotov, V., Chen, G., Povey, D., & Khudanpur, S. (2015). Librispeech: an ASR corpus based on public domain audio books. In *2015 IEEE international conference on acoustics, speech and signal processing (ICASSP),* 5206-5210.
